# Management of Common Bile Duct Stones Encountered During Cholecystectomy in Patients With Previous Gastric Bypass

**DOI:** 10.3389/fsurg.2021.789231

**Published:** 2021-12-08

**Authors:** Agnieszka Popowicz, Susanne Sanamrad, Bahman Darkahi, Rebecka Zacharias, Gabriel Sandblom

**Affiliations:** ^1^Department of Clinical Sciences, Intervention and Technology (CLINTEC), Karolinska Institute, Stockholm, Sweden; ^2^Section of Acute and Trauma Surgery, Karolinska University Hospital, Stockholm, Sweden; ^3^Department of Emergency Medicine, Capio S:t Göran Hospital, Stockholm, Sweden; ^4^Department of Surgery, Enköping Hospital, Enköping, Sweden; ^5^Department of Clinical Science and Education Södersjukhuset, Karolinska Institutet, Stockholm, Sweden; ^6^Department of Surgery, Södersjukhuset, Stockholm, Sweden

**Keywords:** ERCP (cholangiopancreatography), gastric bypass, common bile duct stones, cholangiotomy, transcystic stone extraction

## Abstract

**Background:** Rapid weight loss following gastric bypass (GBP) predisposes to the development of gallstones, and in those who develop gallstone disease there is a high prevalence of common bile duct stones (CBDS). Furthermore, in these patients, CBDS are difficult to extract due to the altered upper gastrointestinal anatomy following GBP. The aim of the present study was to assess outcome after various management methods applied in the counties of Stockholm and Uppsala, Sweden.

**Methods:** Data from the Swedish Register for Gallstone Surgery and ERCP (GallRiks) and the Swedish Obesity Surgery Register (SoReg) were crossmatched to identify all patients who had undergone gallstone surgery after GBP, where CBDS were found at intraoperative cholangiography, in the Stockholm and Uppsala counties 2009–2013. A retrospective review of patient records was performed for all patients identified.

**Results:** In all, 55 patients were identified. These were managed as follows: expectancy (*N* = 11); transgastric ERCP (*N* = 2); laparoscopic choledochotomy (*N* = 3); open choledochotomy (*N* = 5); transcystic stone extraction (*N* = 12); and other approach (*N* = 13). In nine cases, data on management could not be found. There were nine cases of minor postoperative complication. No retained stones were registered. The operation time was longer for transgastric ERCP (*p* = 0.002), and the postoperative stay was longer following open and laparoscopic choledochotomy (*p* < 0.001). There was no statistically significant difference between any of the methods regarding the incidence of postoperative complications (*p* = 0.098).

**Discussion:** Further development of techniques for managing CBDS discovered in patients undergoing cholecystectomy after previous GBP are needed, as well as more comparative studies with greater statistical power.

## Background

Obesity is a well-known risk factor for gallstone formation. Moreover, surgery for obesity increases the risk for gallstone formation more than 3-fold the first 3 years after surgery (1). This is mainly due to an increase in cholesterol stones that are often small, spherical, and hard, and thus tend to migrate to the common bile duct. The rapid increase in bariatric surgery over the last decade has resulted in an almost exponential increase in post-bariatric gallstone disease (1).

The management of common bile duct stones (CBDS) in these patients is also complicated by the fact that the bypassed segment is not readily available for endoscopic or radiographic examination following a Roux-en-Y gastric bypass (2). Several approaches have been suggested for extracting common bile duct stones in patients with a Roux-en-Y limb, including transcystic extraction, laparoscopic cholangiotomy (3), transgastric endoscopic cholangiopancreatography (ERCP) (4–9) and overtube-assisted ERCP (9). Each of these methods are technically complicated, require specific resources, and have certain limitations. At most centers where gallstone surgery is routine, there are usually only one or two methods of management used for CBDS in these patients.

The aim of the present study was to assess outcome after cholecystectomy in patients with a previous history of gastric bypass where CBDS were detected at intraoperative cholangiography.

## Methods

The study was based on data from the Swedish Register for Gallstone Surgery and ERCP (GallRiks) and the Swedish Obesity Surgery Register (SoReg). GallRiks is a nationwide population-based register where all interventions for gallstone disease are registered prospectively, including cholecystectomies and ERCPs. It began in 2005 and now has a coverage of more than 95% of all procedures (10). Variables registered in GallRiks include findings at intraoperative cholangiography.

The SOReg was launched in 2007 to promote clinical quality control, as well as development and research in bariatric surgery. All data in SOReg are registered prospectively by the surgeon performing the procedure. The register had a coverage of 80% in 2008 and more than 97% during the period 2009–2013. It has been validated and shown to have a high degree of accuracy and reliability (11).

Data from the two registers 2007–2013 were crossmatched using the Swedish personal identity number (12). Through crossmatching, all patients who underwent gastric bypass and subsequently cholecystectomy were identified. From this cohort, patients who had undergone cholecystectomy at a unit in the Counties of Stockholm and Uppsala were identified. In cases where intraoperative cholangiography did not show common bile duct stones, the patient was excluded. A retrospective review of the patient records was performed, where additional data on the management of the common bile duct stones were registered according to a predefined protocol.

The following techniques were used to extract the common bile duct stones:

Expectancy. Small stones may be left *in situ* if they can be expected to pass spontaneously. Intraoperative flushing through the cholangiography catheter were also included in this group.

Transgastric ERCP. An intraoperative gastrotomy in the excluded stomach is created in order to access the stomach and duodenum. The endoscope is entered through the gastrotomy and a routine ERCP with sphincterotomy is carried out. After the ERCP, the gastrotomy may either be closed intraoperatively or a gastrostomy may be left to provide access for later procedures.

Laparoscopic choledochotomy. An incision is made in the common bile duct distal to the confluence with the cystic duct. A choledochoscope is introduced into the incision and the stones extracted through the incision. After the stones have been extracted, the incision may either be closed primarily or a T tube may be placed in the incision, preparing for secondary cholangiography.

Open choledochotomy. The procedure is converted to open cholecystectomy, allowing for open choledochotomy and extraction of the common bile duct stones through the incision. The management is similar to laparoscopic choledochotomy, but carried out with open approach.

Laparoscopic transcystic stone extraction. The cystic duct is dilated and the stones are extracted with a Dormia basket or balloon catheter under fluoroscopic guidance.

The study was approved by the Ethics Review Board of Uppsala University (Dnr 2014/036).

### Statistics

Differences in operation time, postoperative stay and time elapsed from bariatric surgery to gallstone surgery between the different methods of management were tested using the Kruskall-Wallis test. Differences in prevalence of common bile duct stones and postoperative complication rates were tested with the chi^2^ test.

## Results

In all, 55 patients who had previously undergone gastric bypass and later underwent gallstone surgery where common bile duct stones were found, were included. The study group included 51 women and four men. Mean age was 42 years (standard deviation 14 years).

The prevalence of common bile duct stones encountered at intraoperative cholangiography in the entire cohort of patients undergoing cholecystectomy was 1,661/18,507 (9.0%). In the subgroup of patients having undergone gastric by-pass, the prevalence was 55/432 (12.7%). This difference was statistically significant (*p* = 0.006).

**Figure 1:**
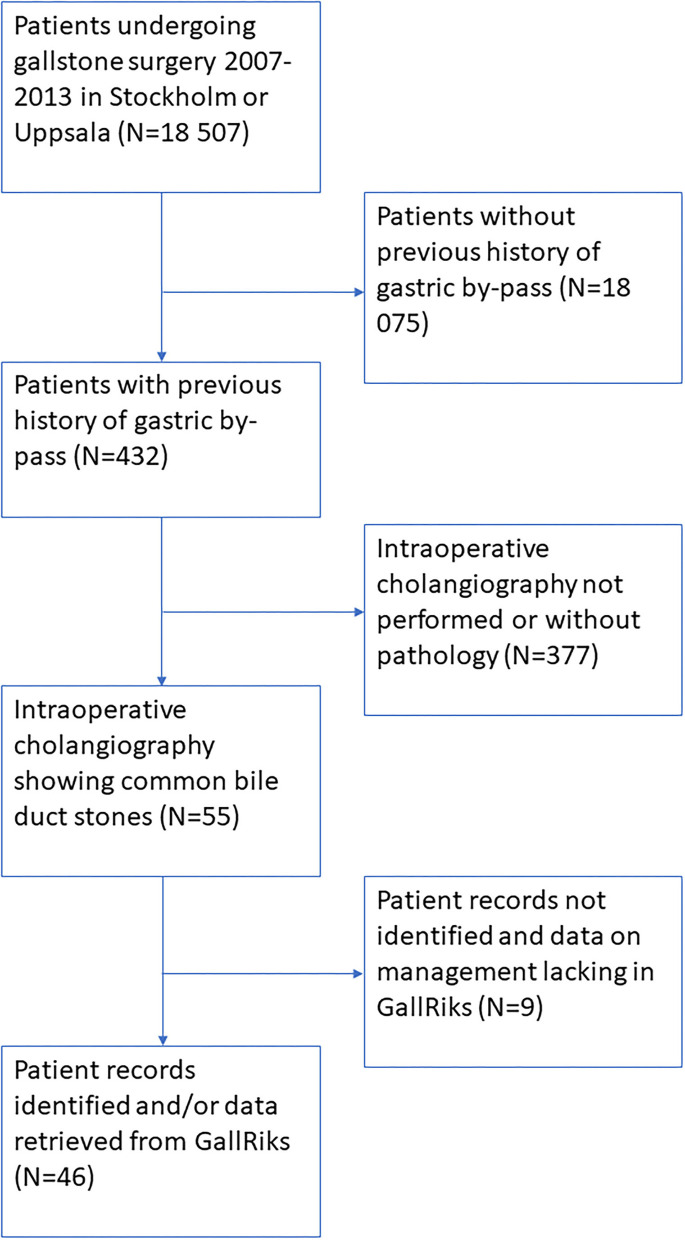
Flow chart.

Details of the cohort are given in Figure 1. The findings at intraoperative cholangiography and treatment outcome are presented in Table 1.

**Table 1:** Treatment outcome.

**Management**	***N***	**Diameter of largest stone >4 mm**	**Median time from gastric by-pass to cholecystectomy, months (range)**	**Median operation time, minutes (range)**	**Median postoperative stay (range)**	**Intra- and postoperative complications**	**Type of complication**
Expectancy	11	1 (9%)	14 (11–37)	87 (30–150)	1 (0–3)	0 (0%)	
Transgastric ERCP	2	1 (50%)	18 (10–26)	349.5 (219–459)	3 (2–4)	0 (0%)	
Laparoscopic cholangiotomy	3	1 (33%)	15 (15–16)	172 (140–212)	6 (3–12)	1 (33%)	Postoperative bile leakage
Open cholangiotomy	5	4 (80%)	30 (6–45)	155 (126–267)	10 (8–17)	0 (0%)	
Laparosopic transcystic stone extraction	12	5 (42%)	13 (2–38)	160 (60–286)	2 (1–8)	5 (42%)	Postoperative abscess, postoperative bile leakage, gastric perforation, postoperative pain (two patients). Retained common bile duct stone requiring delayed ERCP.
Other	13	1 (8%)	15 (5–52)	120 (47–201)	2 (0–5)	2 (15%)	Postoperative pancreatitis, intraoperative bleeding
Data on treatment missing	9	1 (11%)	12 (5–53)	94(47–174)	1 (1–1)	1 (11%)	Trochar incision infection
Total	55	14 (25%)	13 (2–53)	126 (30–459)	1 (0–17)	9 (16%)	

*Fifty-five patients identified. Management: expectancy (N = 11); transgastric ERCP (N = 2); laparoscopic cholangiotomy (N = 3); open cholangiotomy (N = 5); transcystic stone extraction (N = 12); other approach (N = 13). Data on management not available in nine cases. There were nine cases of minor postoperative complication. No retained stones were registered. Operation time was longer for transgastric ERCP, and postoperative stay was longer following open and laparoscopic cholangiotomy. There was no statistically significant difference in the incidence of postoperative complications between groups*.

There were statistically significant differences in operation time (*p* = 0.002) and postoperative stay (*p* < 0.001) between the management groups. The complication rate, however, did not differ between the groups (*p* = 0.098). Time elapsed from bariatric surgery to gallstone procedure did not differ significantly between the groups (*p* = 0.982). One patient having undergone transcystic stone extraction was later diagnosed with a retained common bile duct stone requiring delayed transgastric ERCP.

No major complications were seen in any of the groups, but postoperative bile leakage occurred in one case after laparoscopic choledochotomy, and in one case following transcystic stone extraction. One case of intra-abdominal abscess and one case of gastric perforation was seen after transcystic stone extraction. No retained stones were registered in any of the groups.

## Discussion

The present study, based on data derived from two Swedish population-based registers, does not show any substantial difference in the surgical outcome after the various methods for managing CBDS in patients with a previous history of gastric bypass surgery. Although there were differences in operation time and postoperative stay, there were no substantial differences in the incidence of surgical complications.

The prevalence of common bile duct stones encountered at intraoperative cholangiography was slightly higher in the group of patients having undergone gastric by-pass than those who had not. This is probably explained by the preponderance of cholesterol stones in patients who have undergone bariatric surgery (13). The size and shape of cholesterol stones make them more prone to migrate from the gallbladder to the common bile duct.

One case of retained stones was registered. However, as follow-up times varied between the two surgical centers, it cannot be ruled out that there were cases with late presentation of retained stones, or stones that were managed conservatively. Data on long-term outcome is limited to the timepoint when the review was undertaken.

Although the present study was based on data from two nationwide population-based registers, it did not have sufficient statistical power to show clinically relevant differences in safety and effectiveness of the different approaches. Nevertheless, there were no severe complications in any of the groups. The choice of approach is mainly determined by local tradition, and to our knowledge there is no study showing any technique to be superior to the others.

Although all data in GallRiks and SOReg are registered prospectively, the review of the patient records was carried out retrospectively. This could have lead to a selection bias since the data in the records is not complete for all variables.

Reports of case series on transgastric ERCP have been published recently (4, 7, 14, 15). Although no study has presented a control group, the technique is consistently reported as being safe and feasible in most situations. It is an option in patients having undergone gastric by-pass, but not with a history of biliopancreatic diversion (BPD). The technique requires an experienced endoscopist and care should be taken to make the gastrotomy at enough distance from pylorus to allow proper maneuvering of the duodenoscope. The present study confirms the findings of previous studies, although the median operation time was more than an hour longer than the other techniques. This may be due to lack of experience with this technique when the study was conducted, despite the fact that ERCP is routinely used at most units in the counties of Stockholm and Uppsala. The cannulation frequency is high in transgastric ERCP, the main obstacles is usually to gain access to the excluded stomach (9).

Laparoscopic transcystic stone extraction is feasible in most situations, although it may be difficult to extract large CBDS and stones in patients with a narrow cystic duct (16). In these cases, lithotripsy may be necessary. It may, however, be very difficult to extract CBDS that lie proximal to the confluence. Nevertheless, even if transcystic stone extraction cannot be carried out for all stones, it is less invasive than stone extraction through open or laparoscopic choledochotomy and carries less risk of developing stricture.

Laparoscopic choledochotomy is a technique that enables extraction of most CBDS irrespective of location and size (3). The technique, however, is technically complicated and caries the risk of postoperative bile leakage. It may also lead to bile duct stricture if the choledochotomy is made in a non-dilated bile duct. It also requires appropriate operative room settings and advanced surgical equipment. In the present study, the median postoperative stay was several days longer for patients having undergone open and laparoscopic choledochotomy. This could be due to the fact that a t-tube was left in the bile duct in several cases.

Percutaneous transhepatic biliary drainage followed by image-guided stone extraction has recently been suggested as an approach for managing CBDS in patients following Roux-en-Y gastric bypass (17). The technique may be feasible in selected cases but requires technical skill and good knowledge of the bile duct anatomy.

In conclusion, the techniques applied during the period of study were safe and provided a high stone clearance rate. Transgastric ERCP is gradually becoming the technique of choice for extracting CBDS in patients who have undergone GBP, although the technique has a long learning curve and requires a team that is familiar with the approach. As very few units have the capacity for more than one of the approaches, a randomized controlled trial on patients with CBDS and a history of GBP cannot be expected in the near future. Nevertheless, more comparative studies are necessary to compare the effectiveness and safety of these approaches.

## Data Availability Statement

The raw data supporting the conclusions of this article will be made available by the authors, without undue reservation.

## Ethics Statement

The studies involving human participants were reviewed and approved by Uppsala Ethical Review Board. The patients/participants provided their written informed consent to participate in this study.

## Author Contributions

AP conceptualized the study together with GS and assembled data. SS assembled data and participated in drafting the manuscript. BD assembled data and managed the patients from one of the units. RZ assembled data and developed the surgical technique described. GS had senior responsibility and drafted the manuscript. All authors contributed to the article and approved the submitted version.

## Funding

The study was made possible by a grant from Rut och Rickard Julins Research Foundation.

## Conflict of Interest

The authors declare that the research was conducted in the absence of any commercial or financial relationships that could be construed as a potential conflict of interest.

## Publisher's Note

All claims expressed in this article are solely those of the authors and do not necessarily represent those of their affiliated organizations, or those of the publisher, the editors and the reviewers. Any product that may be evaluated in this article, or claim that may be made by its manufacturer, is not guaranteed or endorsed by the publisher.
